# Anti-inflammatory effects of *Salvia plebeia* R. Br extract in vitro and in ovalbumin-induced mouse model

**DOI:** 10.1186/s40659-016-0102-7

**Published:** 2016-10-05

**Authors:** Hwan-Hee Jang, Su-Yeon Cho, Mi-Ju Kim, Jung-Bong Kim, Sung-Hyen Lee, Mee-Young Lee, Young-Min Lee

**Affiliations:** 1 Functional Food & Nutrition Division, National Institute of Agricultural Science, Rural Development Administration (RDA), Wanju, 565-851 South Korea; 2 Herbal Medicine EBM Research Center, Korea Institute of Oriental Medicine, Daejeon, 305-811 South Korea; 3 Department of Food and Nutrition, Seoul Women’s University, Seoul, 139-774 South Korea

**Keywords:** Anti-inflammatory, Asthma, *Salvia plebeia*

## Abstract

**Background:**

Asthma is an increasing global health problem, and novel strategies to prevent or ameliorate the condition are needed. Here, the effects of 80 % ethanol extracts of *Salvia plebeia* R. Br. (SE) on an induced inflammatory response were investigated.

**Results:**

*Salvia plebeia* R. Br. inhibited production of pro-inflammatory cytokines, such as TNF-α and IL-6, as well as nitric oxide (NO) in LPS-stimulated RAW 264.7 cells. NO and pro-inflammatory cytokine production was suppressed more effectively by SE of the aerial parts (SE-A) than of the roots (SE-R) of *S. plebeia*. In BEAS-2B cells, both SE-A and SE-R inhibited the increase in production of the inflammatory cytokines IL-6 and IL-8. We also investigated the anti-asthmatic effects of SE in an ovalbumin (OVA)-induced BALB/c mouse model. SE-A treatment significantly reduced the number of airway eosinophils, IL-4 and IL-13 levels, mucus production, and inflammatory infiltration, as compared with the corresponding levels in the untreated, OVA-induced mice, and had similar effects to dexamethasone.

**Conclusions:**

*Salvia plebeia* ethanol extract ameliorated the induced inflammatory response in RAW 264.7 and BEAS-2B cells, with more effective inhibition noted for SE-A than for SE-R. SE-A treatment was effective in improving the histopathological changes in the lungs of asthma model mice via modulation of eosinophils and Th2 cytokines. These results suggest that SE-A can be considered as a therapeutic agent that can potentially relieve asthma.

## Background

Asthma is a chronic inflammatory disease characterized by the presence of inflammatory cells in the airway, which can be elicited by a variety of environmental allergens, such as air pollutants and tobacco smoke [1]. Common symptoms in asthma include coughing (especially at night), wheezing, shortness of breath, and chest tightness or pain. These symptoms are caused by chronically hyperactive (contraction of the muscles surrounding the airways) and inflamed airways, which leads to airflow obstruction [2]. Asthma is a major health problem affecting people worldwide; the prevalence of asthma is continually increasing, with substantial associated healthcare expenditures [3, 4]. It is urgent to gain full understanding of the mechanism underlying the development of asthma and to develop preventative and/or ameliorating strategies.

Several drugs are currently available for asthma therapy. Corticosteroids, the most potent nonspecific anti-inflammatory agents, are widely used for improvement of lung function in patients with asthma. However, it is well known that inhaled corticosteroids are limited in their capacity to modify airway remodelling [5]. Therefore, there has been increasing interest in the development of natural drugs with fewer side effects than the currently used agents [6]. Recent studies have indicated that herbal medicines can improve symptoms of asthma, by providing experimental evidence of the inhibition of ovalbumin (OVA)-induced asthma in mouse models of this condition [7, 8].

*Salvia plebeia* R. Br is a biannual grass that is distributed widely across many countries. *S. plebeia* has been shown to have anti-oxidant and anti-inflammatory effects [9]. It has been reported that the compound homoplantaginin isolated from the above-ground parts of *S. plebeia* has a protective effect on oxidative stress-induced hepatocyte injury and inflammatory liver injury [10]. Although *S. plebeia* is traditionally used to treat inflammatory diseases, such as colds, coughs, and mild asthma, the anti-inflammatory and anti-asthma effects of *S. plebeia* have not been reported in detail. In addition, there have been few reports of the anti-inflammatory effects of the different parts of *S. plebeia*.

This study aimed to investigate the anti-inflammatory effects of *S. plebeia* in vitro and in a mouse model of asthma, the OVA-induced mouse model. We hypothesized that *S. plebeia* would show anti-inflammatory effects both in vitro and in vivo and that the activity of the aerial parts and the roots would differ.

## Results

### Effect of *S. plebeia* ethanol extract on lipopolysaccharide-induced inflammatory responses in RAW264.7 Cells

**Fig. 1:**
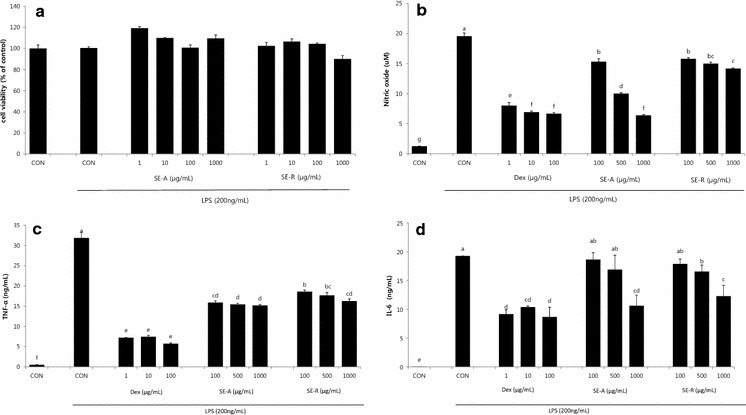
Effect of SE on **a** cell viability, **b** NO production, **c** TNF-α production and **d** IL-6 production in LPS-treated RAW 264.7 macrophages. Values are presented as mean ± SE (n = 3). Means with the* same letters* are not significantly different at P < 0.05 using Duncan’s multiple-range test

To investigate the anti-inflammatory effect of *S. plebeia* (SE), SE was treated on lipopolysaccharide-induced inflammatory responses in RAW 264.7 cells. A 3-(4,5-dimethylthiazol-2-yl)-2,5-diphenyltetrazolium bromide (MTT) assay showed that the 80 % ethanol extract ofSE had no significant effect on RAW 264.7 cell viability at a concentration of 1000 μg/mL. Treatment of RAW 264.7 macrophages with LPS (200 ng/mL) caused a significant increase in NO, TNF-α, and IL-6 production. However, both the extract of the aerial parts (SE-A) and of the roots (SE-R) of *S. plebeia* markedly inhibited the LPS-induced production of NO, TNF-α, and IL-6, in a dose-dependent manner. In particular, NO and pro-inflammatory cytokine production was suppressed more effectively by SE-A than by SE-R (Fig. 1).

**Fig. 2:**
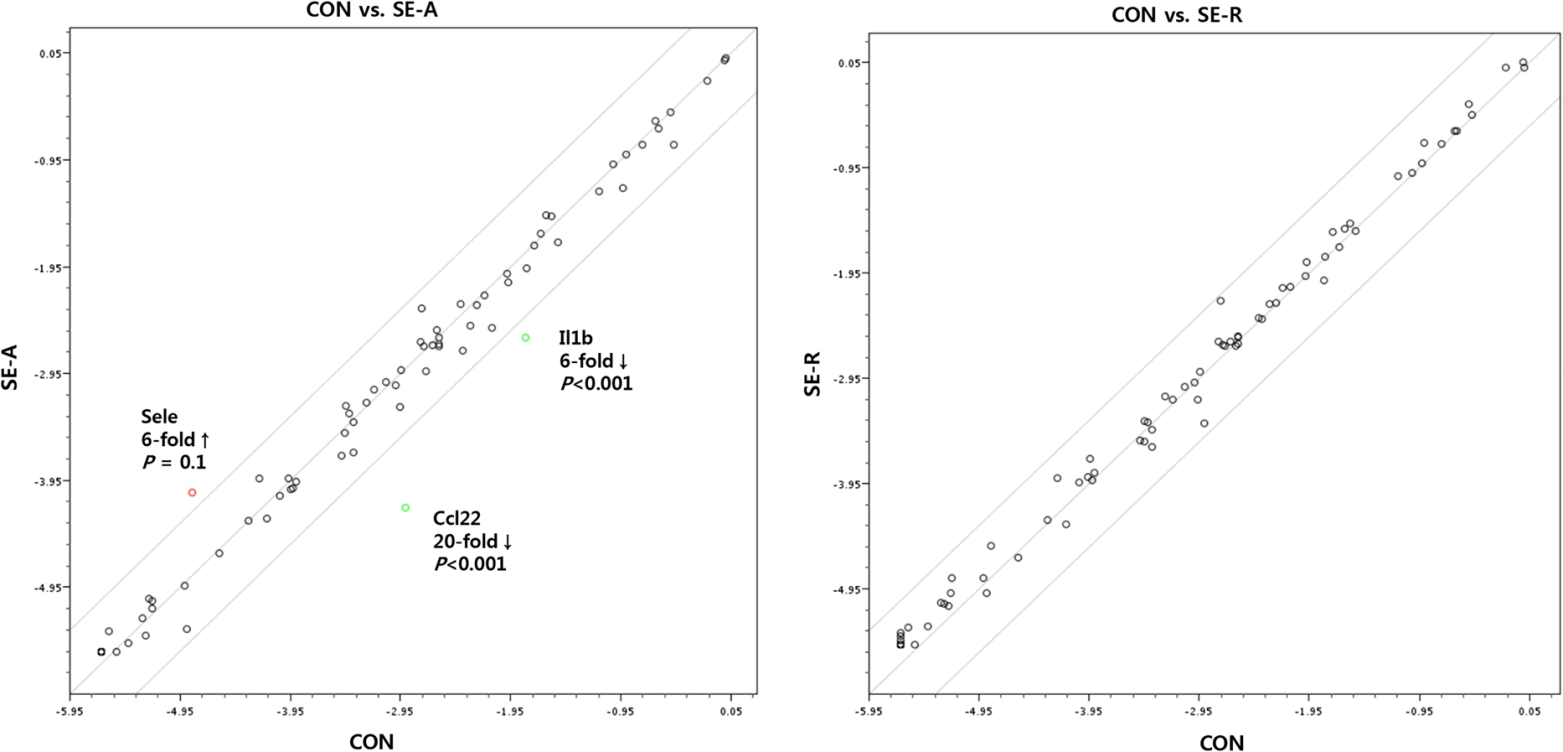
Focused quantitative RT-PCR analysis of inflammatory responses in RAW 264.7 cells exposed to SE-A or SE-R. Both SE-A and SE-R were used at concentrations of 1000 μg/mL. *Lines* above and below the centre regression *lines* indicate fourfold changes in gene expression (three replicates)

To identify inflammatory genes modulated by SE, we compared the gene expression profiles of LPS-stimulated RAW 264.7 and LPS-stimulated RAW 264.7 cells treated with SE-A or SE-R using the Mouse Inflammatory Response & Autoimmunity RT^2^ Profiler PCR array (SABiosciences, Frederick, MD, USA). Quantitative RT-PCR array experiments showed that expression of most genes in LPS-stimulated RAW 264.7 cells remained unchanged, by at least a fourfold margin, after treatment with either of the SE (Fig. 2). However, SE-A treatment modulated three genes associated with inflammatory signalling in RAW 264.7 cells. Expression of the genes encoding chemokine (C–C motif) ligand 22 (*Ccl*22) and interleukin 1 beta (*Il1*b) was markedly reduced in SE-A-treated RAW 264.7 cells. Although not statistically significant, the selectin, endothelial cell (SELE) protein, was increased sixfold by SE-A treatment as compared with untreated, LPS-stimulated RAW 264.7 cells (P = 0.10).

### Effect of SE on TNF-α/LPS-induced inflammatory responses in BEAS-2B cells

**Fig. 3:**
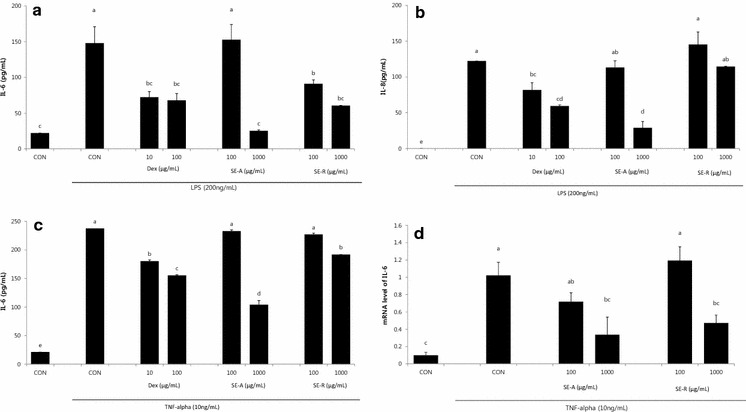
Effect of SE on **a** IL-6 production and **b** IL-8 production in LPS-treated BEAS-2B cells and on **c** IL-6 production and **d** *IL6* mRNA levels in TNF-α-treated BEAS-2B cells. Values are presented as mean ± SE (n = 3). Means with the* same letters* are not significantly different at P < 0.05 using Duncan’s multiple-range test

We treated BEAS-2B human bronchial epithelial cells with TNF-α (10 ng/mL) or LPS (200 ng/mL) to stimulate inflammatory mediator production and assessed the effect of SE. SE had no significant effect on BEAS-2B cell viability up to a dose of 1000 μg/mL (data not shown). In LPS-stimulated BEAS-2B, both SE-A and SE-R effectively prevented the increase in IL-6 and IL-8 production, as compared to treatment with LPS alone (Fig. 3). The production of IL-6 was significantly increased by TNF-α and significantly inhibited by SE-A and SE-R in a dose-dependent manner.

### Effect of SE-A on inflammatory cell numbers in mouse bronchoalveolar lavage fluid (BALF)

**Fig. 4:**
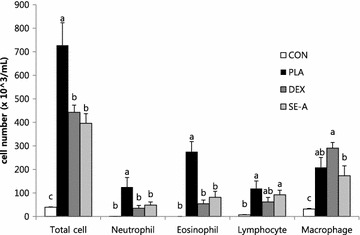
Effects of SE on the recruitment of inflammatory cell numbers in the BALF of mice. Cells were isolated by centrifugation and stained with Diff-Quik staining reagent. Cell numbers were determined under a light microscope. Values are expressed as mean ± SE (n = 8). Means with the* same letters* are not significantly different at P < 0.05 using Duncan’s multiple-range test

Eighteen hours after the final intranasal OVA or PBS challenge, BALF from the lungs was collected to determine the levels of recruited inflammatory cells. As shown in Fig. 4, the numbers of total cells in the BALF obtained from the OVA-challenged group were significantly higher than those of CON group. OVA challenge increased inflammatory cells, such as neutrophils, eosinophils, lymphocytes, and macrophages. On the other hand, SE-A treatment significantly decreased the total number of cells, and the numbers of neutrophils and eosinophils, in BALF as compared with the PLA group.

### Effect of SE on inflammatory cell infiltration and mucus production in lung tissue

**Fig. 5:**
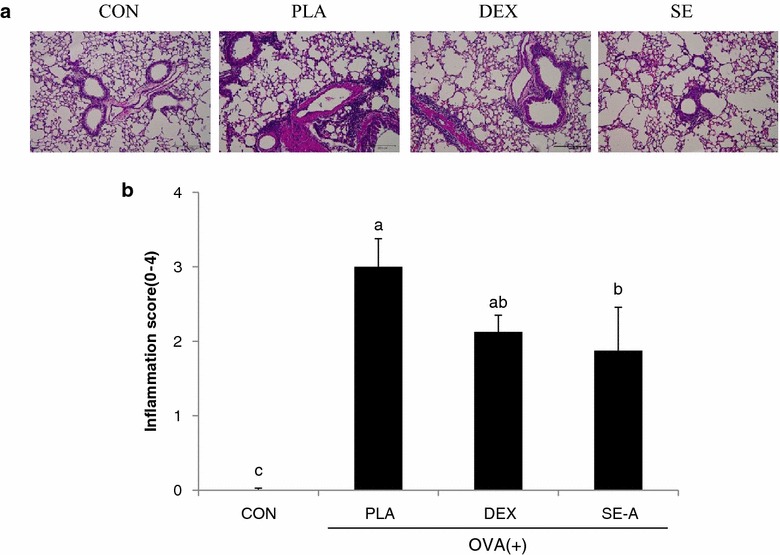
Effects of SE on the recruitment of inflammatory cells in mouse lung tissue. **a** Histological examination of lung tissue was performed 24 h after the last OVA challenge. Lung tissues were fixed, sectioned at 4-µm thickness, and stained with H&E solution. **b** Scoring of the extent of inflammation by quantitative analysis of inflammatory cell infiltration in lung sections. Values are expressed as mean ± SE (n = 8). Means with the* same letters* are not significantly different at P < 0.05 using Duncan’s multiple-range test

To investigate the inhibitory effect of SE-A treatment on inflammatory infiltration and mucus production, we analyzed the histology of lung tissue using hematoxylin and eosin (H&E) and periodic acid-Schiff (PAS) staining, respectively. The number of infiltrated cells was increased in the lungs of the PLA group, as compared with the CON group, as characterized by observation of an intense inflammatory infiltrate. On the other hand, inflammation was alleviated by SE-A treatment, as indicated by the reduced surface area of inflammatory infiltration observed in lungs from mice in the SE-A group (Fig. 5).

**Fig. 6:**
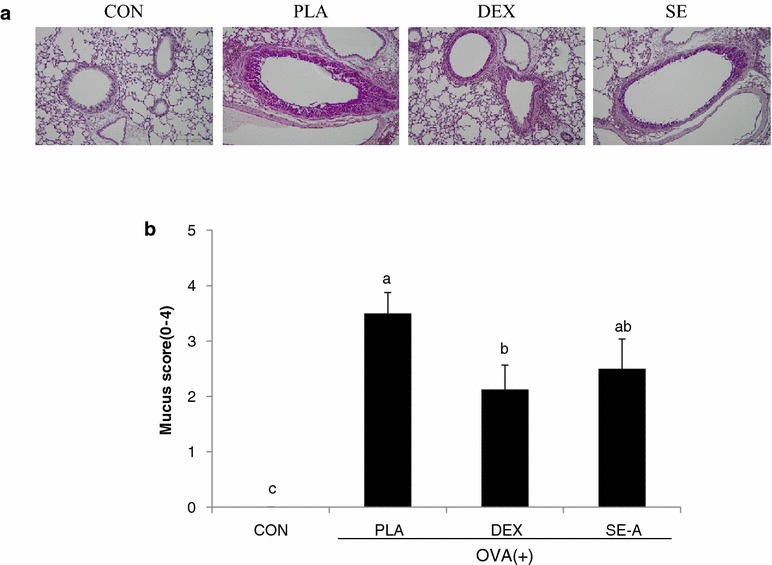
Effects of SE on mucus production in lung tissue. **a** Histological examination of mucus secretion in lung tissue was performed 24 h after the last OVA challenge. Lung tissues were fixed, sectioned at 4-µm thickness, and stained with periodic acid Schiff (PAS) reagent to assess mucus production (magnification ×100 and 200). **b** Scoring of mucus was quantified according to the percentage of PAS-positivity among all epithelial cells. Values are expressed as mean ± SE (n = 8). Means with the* same letters* are not significantly different at P < 0.05 using Duncan’s multiple-range test

Mucus overproduction was observed in the bronchial airway of the PLA group compared with the CON group. The score of mucus production in the SE-A group showed a trend towards reduction, as compared to the PLA group, which approached, but did not attain, statistical significance (Fig. 6).

### Effect of SE on cytokine levels in BALF

**Fig. 7:**
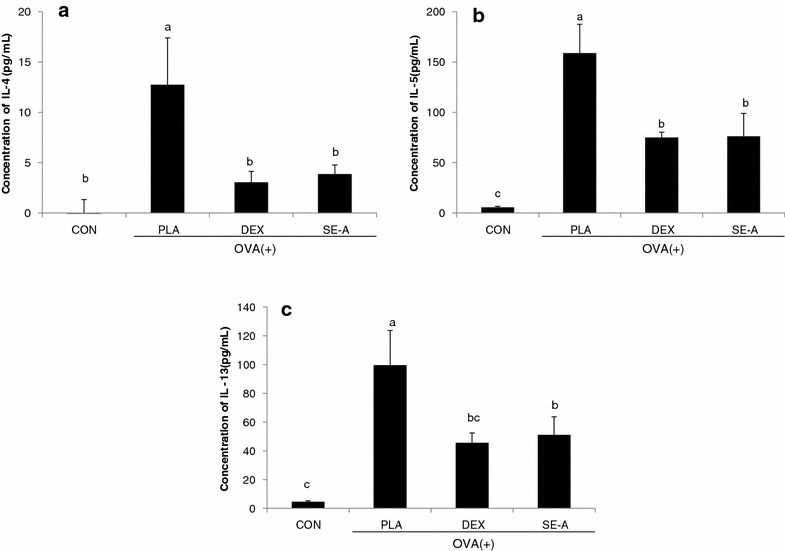
Effects of SE-A on cytokine levels in BALF. BALF was collected 24 h after the final OVA challenge in mice. Each sample was analyzed using ELISAs. **a** IL-4, **b** IL-5 and **c** IL-13. Values are expressed as mean ± SE (n = 8). Means with the* same letters* are not significantly different at P < 0.05 using Duncan’s multiple-range test

The levels of Th2 cell-derived cytokines, such as IL-4, IL-5, and IL-13 were examined in the BALF of OVA-stimulated BALB/c mice. Th2 cytokine levels in the BALF of OVA-stimulated BALB/c mice were significantly higher than those in the PBS-stimulated group. However, the SE-A-treated group showed significantly reduced IL-4, IL-5, and IL-13 levels (Fig. 7).

## Discussion

The present study investigated the anti-inflammatory effects of *S. plebeia* both in vitro and in vivo. Both the extract of the aerial parts (SE-A) and of the roots (SE-R) of *S. plebeia* markedly inhibited the LPS-induced production of NO, TNF-α, and IL-6, in a dose-dependent manner. Jung et al. [9] showed that the ethanol extract of the whole plant of *S. plebeia* significantly decreased the levels of NO and iNOS in LPS-stimulated RAW 264.7 cells. However, we used extracts of the aerial and root parts of *S. plebeia* separately, and found that the aerial parts were more effective in ameliorating allergic inflammatory responses.

In quantitative RT-PCR array experiments, LPS-stimulated RAW 264.7 cells treated with SE-A modulated three genes associated with inflammatory signalling in RAW 264.7 cells, by at least a fourfold margin. Interestingly, the gene encoding chemokine (C–C motif) ligand 22 (*Ccl*22), which induces the selective migration of Th2 cells, but not of Th1 cells, by triggering CCR4 [11], was markedly reduced in SE-A-treated RAW 264.7 cells. The gene encoding interleukin 1 beta (*Il1*b) was also reduced sixfold by SE-A treatment in LPS-stimulated RAW 264.7 cells. *Il1*b has been known as a pro-inflammatory cytokine that has been implicated in driving the inflammatory process in various disease states, including asthma [12]. Although not statistically significant, the selectin, endothelial cell (SELE) protein, which has been noted to play important roles in the production of allergic inflammation [13], was increased sixfold by SE-A treatment as compared with untreated, LPS-stimulated RAW 264.7 cells. These data indicated that SE-A showed improved inflammatory responses through modulation of the expression of the relevant genes.

Next, we investigated the anti-inflammatory effect of both SE-A and SE-R in stimulated BEAS-2B human bronchial epithelial cells. BEAS-2B was treated with TNF-α or LPS to stimulate inflammatory mediators. Upon stimulation with LPS, TNF-α, or IL-4, BEAS-2B cells are reported to secrete pathologically elevated levels of cytokines and chemokines, including IL-6, IL-8, eotaxin, and RANTES [14, 15]. Our data showed that SE-A and SE-R had an inhibitory effect on the production of cytokines, including IL-6 and IL-8, in stimulated cells, with SE-A showing more effective inhibition. In addition, in a real-time PCR assay, SE-A also effectively decreased the levels of *IL6* mRNA in TNF-α-stimulated BEAS-2B cells compare to SE-R.

This in vitro study showed that SE-A and SE-R had an inhibitory effect on inflammatory responses in stimulated cells, with SE-A showing more effective inhibition. Inflammation related genes was regulated more effectively by SE-A than of SE-R. Thus, we further studied the anti-inflammatory effects of SE-A in vivo to confirm the physiological relevance of our in vitro findings.

In vivo study was performed using OVA-induced asthma model mice. The major feature of asthma is airway inflammation, which predominantly involves eosinophils, macrophages, and mast cells [16]. Important cell types in airway inflammation are eosinophils and leukocytes, which are not only present in the airway wall, but are also present in large numbers in the sputum and BALF [17]. To assess inflammatory cell numbers, BALF was collected from the lungs of OVA-stimulated BALB/c mice. The numbers of total cells, neutrophils, eosinophils, lymphocytes, and macrophages in BALF were significantly increased in the placebo (PLA) group compared with the control (CON) group. SE-A treatment significantly decreased the total number of cells, and the numbers of neutrophils and eosinophils, in BALF as compared with the PLA group. SE-A mainly decreased the numbers of eosinophils, which reflect asthmatic activity and the severity of asthma [18, 19]. However, the numbers of lymphocytes and macrophages did not significantly decrease with SE-A treatment. These effects were similar between the SE-A and dexamethasone (DEX) groups. These results were confirmed by histological analysis, which showed that SE inhibited inflammatory cell infiltration and mucus hypersecretion.

To investigate the inhibitory effect of SE-A treatment on inflammatory infiltration and mucus production, we analyzed the histology of lung tissue using H&E and PAS staining, respectively. The number of infiltrated cells was increased in the lungs of the PLA group, as characterized by an intense inflammatory infiltrate, as compared with the CON group. On the other hand, inflammation was alleviated by SE-A treatment, as indicated by the reduced surface area of inflammatory infiltration observed in lungs from mice in the SE-A group.

Mucus overproduction was observed in the bronchial airway of the PLA group compared with the CON group. The mucus production in the SE-A group tended to be reduced as compared to the PLA group. Histopathological alterations, such as goblet cell hyperplasia, mucus hypersecretion, and infiltration of inflammatory cells in lung tissue, are observed in asthmatic conditions [20]. IL-4 and IL-13 play critical roles in IgE switching within B cells, which triggers mucus hypersecretion and goblet cell hyperplasia, whereas IL-5 is essential for the activation and survival of eosinophils, as well as in the development of airway hyper responsiveness [21]. Similar alterations, including histopathological changes, were seen in the lung tissue of OVA-induced mice. However, the extent of these alterations was significantly decreased in SE-A-treated mice, similar to that observed in DEX-treated mice, which were used as the positive control. These data were in agreement with the results obtained for Th2 cytokines in BALF. Based on these findings, we propose that SE-A attenuates the inflammatory response in OVA-induced allergic asthma via modulation of eosinophils and Th2 cytokines.

The levels of Th2 cell-derived cytokines, such as IL-4, IL-5, and IL-13 were examined in the BALF of OVA-stimulated BALB/c mice. Th2 cytokine levels in the PLA group were significantly higher than those in the CON group. However, the SE-A-treated group showed significantly reduced IL-4, IL-5, and IL-13 levels. IL-4 and IL-13 can be produced by a variety of cell types of the innate immune system, which plays a crucial role during asthma [1]. IL-13 induces the pathophysiological features of asthma in a manner that is independent of IgE and eosinophils [22]. Infiltration of eosinophils into the airways is linked to the production of IL-5, which is important for eosinophil proliferation, activation, and migration [23]. Our data show that SE inhibits the pulmonary accumulation of eosinophils, in parallel with a decrease in the IL-5 level in BALF. These results suggest that SE-A plays a key role in blocking mucus secretion and recruitment of eosinophils in the lungs, partially via inhibition of IL-4-, IL-5-, and IL-13-dependent pathways.

Several studies have shown that the aerial parts of *S. plebeia* are rich in flavonoids, such as luteolin, nepitrin, quercetin, rosmarinic, and homoplantaginin [24, 25]. Jin et al. reported that luteolin-7-O-glucoside isolated from the leaves and branches of *Ailanthus altissima* has an anti-asthmatic effect through the downregulation of Th2 cell-derived cytokine expression in an OVA-induced asthma model [26]. The compound homoplantaginin, which is the main flavonoid from *S. plebeia,*is known to have inhibitory effects on inflammation [27]. He et al. reported that homoplantaginin ameliorated palmitic acid-induced endothelial inflammation by suppressing toll-like receptor-4 and NLRP3 pathways, and restoring nitric oxide generation [28]. It is expected that the positive effects of SE treatment can likely be attributed to the flavonoids, such as luteolin and homoplantaginin, within SE.

## Conclusions

In conclusion, the ethanol extract of *S. plebeia* ameliorated the inflammatory response stimulated by LPS and/or TNF-α in RAW 264.7 and BEAS-2B cells, with more effective inhibition noted for SE-A than for SE-R. SE-A treatment was effective in improving histopathological changes in the lungs in asthma model mice via modulation of eosinophils and Th2 cytokines. Further studies are required to determine the specific mechanisms of action of this extract of *S. plebeia*.

## Methods

### In vitro studies

#### Plant material and extract preparation

The aerial parts and roots of *S. plebeia* were purchased from material harvested from a natural population on a farm in the Paju area of the Gyeonggi-do (South Korea).

After a multiple-step cleaning process and drying, 300 g of the different parts of *S. plebeia* was extracted twice with 10 volumes of 80 % ethanol at room temperature for 16 and 3 h, respectively. The extracts of the different parts of *S. plebeia* were filtered through No. 6 filter paper (Advantec Co., Tokyo, Japan) and were concentrated until dry by sequential use of a rotary evaporator (EYELA N-1000, Riakikai Co., Ltd., Tokyo, Japan) at 30 °C. Then, each of the 80 % ethanol filtrates were frozen and lyophilized (PVTFD 10R, Ilsin Lab, Yangju, Korea). The final lyophilized extracts were stored at −70 °C until required for experimental use.

#### Cells and culture

Murine macrophage RAW264.7 cells (Korean Cell Line Bank, Seoul, Korea) and human bronchial epithelial BEAS-2B cells (ATCC, Manassas, VA, USA) were used for in vitro experiments. These cells were cultured in DMEM (Gibco, Rockville, IL, USA) supplemented with 10 % heat-inactivated fetal bovine serum (Gibco, Rockville, IL, USA) and penicillin–streptomycin solution (100 units/mL penicillin and 100 μg/mL streptomycin; HyClone Laboratories Inc., South Logan, UT, USA). BEAS-2B cells were cultured in BEGE medium with a Bullet Kit (Lonza, Walkersville, MD, USA). All cells were grown in a 5 % CO_2_-humidified atmosphere at 37 °C.

#### Preparation of LPS and TNF-α solution

Lipopolysaccharide (LPS) was dissolved in phosphate-buffered saline at 1 mg/mL and stored at −20 °C. This solution was filtered through a 0.22-μm membrane before use. A fresh stock solution of TNF-α (200 μg/mL) was prepared in phosphate-buffered saline and was added directly to cell culture medium. LPS and TNF-α were treated at a final concentration of 200 and 10 ng/mL individually to stimulate cell cultures [29].

#### Cell cytotoxicity assay

Cell cytotoxicity was assessed using the MTT assay. After the incubation period, cells were added to 100 μL of 5 mg/mL thiazolyl blue tetrazolium bromide (Sigma, St. Louis, MO) solution/well and were incubated further for 4 h in a humidified atmosphere (37 °C in 5 % CO_2_). The medium was replaced with 1 mL dimethyl sulfoxide (DMSO). The absorbance was measured at 540 nm in a microplate reader (Molecular Devices Inc., Sunnyvale, CA). Cell cytotoxicity was expressed as percentage values compared with the negative, phosphate-buffered saline (PBS) control, which was considered to represent 100 % cell viability.

#### Nitric oxide measurement

The anti-inflammatory properties of the aerial parts and roots from *S. plebeia* were determined in LPS-stimulated RAW264.7 cells. RAW264.7 cells were incubated with 200 ng/mL of LPS in the presence of ethanol extracts of the aerial parts or roots of *S. plebeia* (100–1000 μg/mL) for 24 h. Briefly, equal volumes of incubation medium supernatant and Griess reagent were allowed to react for 15 min, and the nitrite content was measured by determining absorbance of the mixture at 540 nm. The nitrite concentration in the sample was calculated using a standard curve prepared with NaNO_2_.

#### Measurement of inflammatory cytokine levels

TNF-α, IL-6, and IL-8 cytokine levels in the RAW264.7 and BEAS-2B cell culture media were measured using an enzyme-linked immunosorbent assay (ELISA), according to the manufacturer’s protocols (BD Biosciences. San Diego, CA, USA).

#### Real-time quantitative reverse transcription-PCR

Total RNA was isolated from RAW264.7 or BEAS-2B cells using the RNeasy Mini Kit (Qiagen, Valencia, CA, USA), and RNA integrity (RIN > 9.0) was assessed using a Bioanalyzer 2100 (Agilent Technologies, Santa Clara, CA, USA). The Mouse Inflammatory Response & Autoimmunity PCR array (SABiosciences, Frederick, MD, USA) were used to profile the genes differentially expressed in RAW 264.7 cells, according to the manufacturer’s instructions. The complete list of genes assayed on the array is provided on the manufacturer’s website (http://www.sabiosciences.com/PCRArrayPlate.php). For each plate, 0.5 µg of RNA was converted to double-stranded cDNA using the RT^2^ first strand synthesis kit (Qiagen, Valencia, CA, USA). After mixing this with the SABiosciences RT^2^ qPCR master mix, the cDNA was pipetted into the 96-well profile plate and amplified on CFX96TM real-time pcr detection system (BIO-RAD). Data were normalized using β-actin as an endogenous control and fold-changes in expression were calculated using SABiosciences online software (http://pcrdataanalysis.sabiosciences.com/pcr/arrayanalysis.php). To observe the maximum differential expression, both SE-A and SE-R were used at concentrations of 1000 μg/mL. For *Il6*, expression was detected by using the CFX96 real time system (Bio-Rad) with *β-actin* as control. The primer sequences are as follows, *β*-*actin* forward: 5′-GTGGGGCGCCCCAGGCACCA-3′, *β*-*actin* reverse: 5′-CTCCTTAATGTCACGCACGATTTC-3′, *IL6* forward: 5′-TGGCTGAAAAAGATGGATGCT-3′, *IL6* reverse: 5′-AACTCCAAAAGACCAGTGATGATTT-3′.

### In vivo studies

#### Animals and ethical approval

The experimental design was approved by the Institutional Animal Care and Use Committee (IACUC) of the National Academy of Agricultural Science (reference number: NAAS-1307).

Six-week-old female BALB/c mice (15–19 g) were purchased from Central Lab. Animal Inc. (Seoul, Korea). Mice were housed in Plexiglas cages and maintained in an air-conditioned room at 23 ± 3 °C under an automatic lighting schedule (12-h light/dark cycle). The mice were allowed free access to water and a laboratory diet (Purina Inc., St Louis, MO, USA) for the experimental period.

#### Sensitization and inhalational exposure

**Fig. 8:**
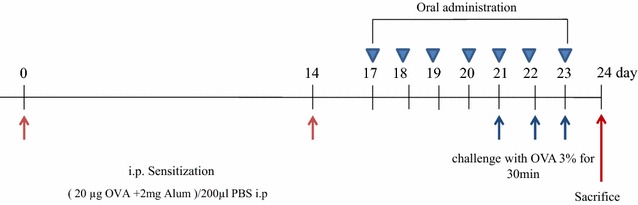
Mouse model of allergic asthma. Mice were sensitized with an intraperitoneal (i.p.) injection of 20 µg of ovalbumin (OVA) emulsified in 2 mg aluminium hydroxide in a total volume of 200 µL in phosphate-buffered saline (PBS) per animal on days 0 and 14. The challenge was administered by inhalation of nebulized 3 % OVA for 30 min on days 21, 22, and 23. Oral treatment, consisting of 3 mg/kg of dexamethasone or 100 mg/kg of SE-A, was administered from day 17 to day 23 of the protocol

A schematic presentation of sensitization and inhalation exposure is shown in Fig. 8. Thirty-two mice were divided into four groups; groups I (control [CON]), II (placebo [PLA]), III (dexamethasone 3 mg kg^−1^ day^−1^ [DEX]), and IV (SE-A 100 mg kg^−1^ day^−1^), with each group including eight mice. Mice in Groups II, III, and IV were sensitized with an intraperitoneal (i.p.) injection of 20 µg of OVA emulsified in 2 mg aluminium hydroxide in a total volume of 200 µL in PBS per animal on days 0 and 14. The challenge was administered by inhalation of nebulized 3 % OVA for 30 min on days 21, 22, and 23. Oral treatment, consisting of 3 mg/kg of dexamethasone (group III), or 100 mg/kg of SE-A (group IV), was administered daily from day 17 to day 23 of the protocol. CON and PLA mice were orally treated with PBS. Mice in the control group (group I) received PBS without OVA on days 0 and 14 and aerosolized saline without alum for 30 min on days 21, 22, and 23.

#### Collection of bronchoalveolar lavage fluid and lung samples

On the day after the last challenge, mice were euthanized with a solution of Zoletil (250 mg/5 cc, Virbac, Carros, France) and Rumpun (2 %, Bayer, Leverkusen, Germany). Bronchoalveolar lavage (BAL) was performed four times by intratracheal instillation of 250 µL of phosphate-buffered saline. The BAL fluid (BALF) was centrifuged, and the supernatants were used for cytokine measurements. Cell pellets were resuspended in 1 mL of PBS and used for total and differential cell counts. The total cell number in BALF samples was counted using a hemocytometer. Numbers of infiltrated inflammatory cells, stained with Diff-Quick, were quantified by microscopy.

#### Histopathological analysis

After BALF was obtained, lungs of the mice were removed by dissection and fixed in 10 % formalin, and then embedded in paraffin. Sections were cut at 4-µm thickness and stained with hematoxylin and eosin (H&E) for the evaluation of inflammatory cell infiltration, or with periodic acid-Schiff (PAS) for the identification of goblet cells. The slides were examined by a treatment-blinded pathologist. The inflammatory score was graded on the following scale: 0, none; 1, minimal; 2, mild; 3, moderate; 4, severe. Hyperplasia of the goblet cells was quantified according to the percentage of PAS-positivity among all epithelial cells: 0, none; 1, <25 %; 2, 25–50 %; 3, 50–75 %; 4, >75 % [23]. Scoring of inflammatory cells and goblet cells was performed in at least 3 different fields for each lung section.

#### Measurement of inflammatory cytokine levels

IL-4, IL-5, and IL-13 levels in BALF were determined by ELISA according to the manufacturer’s protocols (R&D system, Minneapolis, MN, USA).

#### Statistical analysis

Data were expressed as mean ± SE (or SD) for each group. All statistical analyses were performed with SAS 9.2 (SAS Institute; Cary, NY, USA). Results were analyzed by one-way analysis of variance. When a significant difference was indicated, a Duncan’s multiple-range test was performed to determine significant differences among the groups. A *P* value <0.05 was considered statistically significant.

## Abbreviations

LPS: lipopolysaccharide
NO: nitric oxide
OVA: ovalbumin
PAS: periodic acid-Schiff
PBS: phosphate buffered saline
PCR: polymerase chain reaction
qPCR: quantitative real time polymerase chain reaction
RNA: ribonucleic acid
RT-PCR: real time reverse transcription polymerase chain reaction
Th1: type 1 helper T cell
Th2: type 2 helper T cell
TNF-α: tumournecrosis factor-α
